# An Assessment of the Acceptability of Cervical Cancer Education Intervention Materials Among Healthcare Providers in Ghana

**DOI:** 10.1007/s13187-024-02501-1

**Published:** 2024-09-10

**Authors:** Juliet Bonnah, Michelle S. Williams

**Affiliations:** Department of Global and Community Health, College of Public Health, George Mason University, 4400 University Boulevard, MS 5B7, Fairfax, VA 22030 USA

**Keywords:** Cervical cancer prevention, Health communication, Ghana

## Abstract

Cervical cancer is currently the second leading cause of cancer death among women in Ghana. Previous studies have identified lack of awareness, lack of perceived susceptibility, and stigmatizing beliefs as significant sociocultural barriers to cervical cancer screening among Ghanaian women. The purpose of this study was to assess the acceptability of evidence and theory-based, culturally relevant cervical cancer education intervention materials among Ghanaian healthcare providers. Central-location intercept questionnaires were completed by providers (*n* = 60) in the Greater Accra region of Ghana. Providers reviewed a poster, an audio message, and a brief educational video. The variables assessed included the reaction to the materials, the ability of the materials to attract the attention of the intended audience, the ability of the materials to communicate the main point of the cancer education message, and the reaction to cultural characteristics of the materials. The mean age of the providers (*n* = 60) was 30.6 years, and the majority (70.8%) were females. Most of the providers had a positive general reaction to the poster, audio message, and video. The majority found the materials to be motivating. Most of the providers found the information in the materials to be attention-getting, interesting, useful, direct/to the point, and related to someone like them. Very few providers (5%) indicated that they were confused by the images or messages used in the materials. The culturally relevant cervical cancer education materials were acceptable to Ghanaian healthcare providers. These materials may be effective in shared decision-making for cervical cancer screening.

## Introduction

The global cervical cancer mortality rate has declined significantly over the last 20 years [1–4]. However, women in Low- and Middle-Income Countries (LMICs), such as Ghana, continue to experience elevated rates of cervical cancer mortality [1, 3, 5]. Cervical cancer can be prevented if precancerous lesions, cervical intraepithelial neoplasia (CIN), are detected and treated before they progress [6, 7]. Furthermore, early-stage cervical cancer is highly treatable, even in low resource settings [8, 9]. In Ghana, the incidence of CIN and early-stage cervical cancer are low in comparison to the incidence of late stage, invasive cervical cancer [10–13]. Cervical cancer also causes an excessively high age-standardized rate of Years Lived with Disability (YLDs) among Ghanaian women (46/100,000 women in Ghana vs. 28/100,000 women globally) [4, 10].

The cost of the treatment for late-stage cervical cancer is prohibitive for many Ghanaian women [10, 11]. Therefore, the early detection and treatment of CIN and early-stage cervical cancer should be the paramount goal of cervical cancer prevention and control in Ghana. However, less than 4% of Ghanaian women adhere to the World Health Organization’s cervical cancer screening guidelines [14]. Previous studies have shown that there are significant sociocultural barriers to cervical cancer screening among Ghanaian women, including (1) the lack of awareness about cervical cancer and cervical cancer screening, (2) the stigmatization of women with cervical cancer, and (3) the minimization of perceived susceptibility to cervical cancer due to stigmatizing beliefs [15–18]. These sociocultural barriers were found to be common among Ghanaian women of all education levels [16].

Evidence- and theory-based cervical cancer education interventions implemented in LMICs have been shown to increase cervical cancer awareness and intentions for getting screened [19–21]. Evidence has shown that health education interventions are most effective when they are culturally sensitive by being tailored to the surface structure and deep structure characteristics of the target audience [22–25]. In addition, to be effective, health promotion messages must be delivered through channels that will reach the target audience [26, 27]. Healthcare providers are an important channel for the dissemination of cancer education materials [28]. Currently, in Ghana, there is a significant lack of culturally sensitive cervical cancer education interventions that could be used by healthcare providers to engage their patients in shared decision-making about cervical cancer screening. A culturally sensitive cervical cancer education intervention was developed to fill that gap.

The goal of this study is to assess healthcare providers’ perceptions of culturally sensitive cervical cancer education intervention messages and materials. Sixty healthcare providers in the Greater Accra Region of Ghana participated in the study.

## Methods

### Design, Setting, and Participant Selection

A cross-sectional study was conducted in the Greater Accra Region of Ghana over a 2-week period during the Summer of 2023. A purposive sampling strategy was used to recruit healthcare providers to participate in the study. A sample size calculation was not warranted since the goal of the study was to assess the effectiveness of a health communication technique and was not intended to make interferences about a population [29]. We aimed to recruit 60 participants. A member of the research team who was a Ghanaian healthcare provider recruited participants and collected the data. The participants were recruited from health centers, polyclinics, district hospitals, and teaching hospitals through direct-person-to-person contact. Healthcare providers who expressed interest in participating in the study completed an eligibility screening assessment. Healthcare providers were eligible to participate in the study if they were at least 18 years old; currently working at a healthcare facility; could speak, hear, and comprehend English and Twi; and had no hearing, speaking or cognitive difficulties.

### Message Testing

**Fig. 1:**
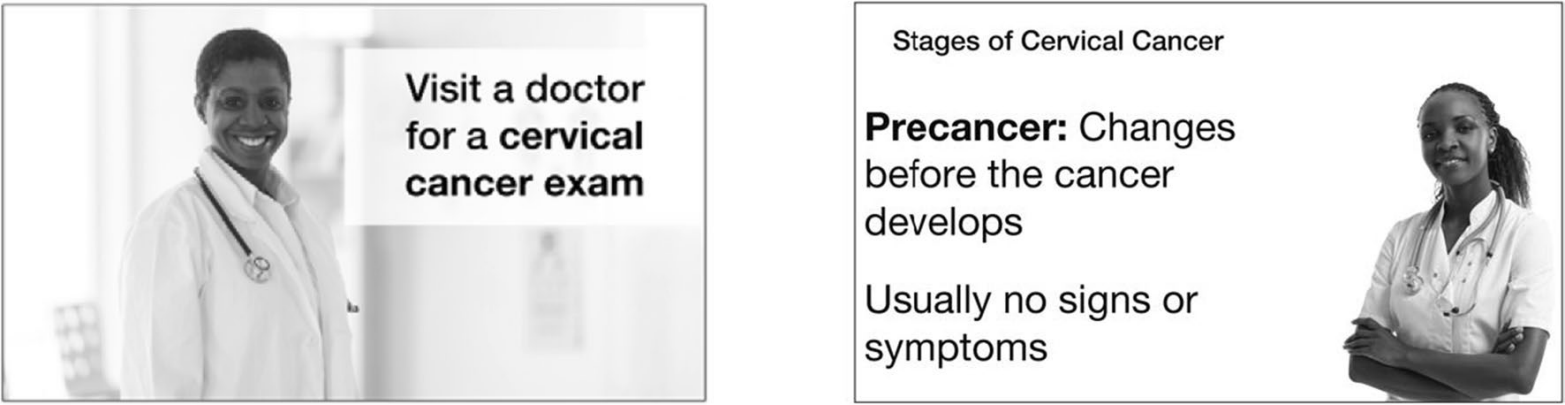
Sample of the storyboard from the cervical cancer education video

The cancer education intervention materials that were tested included a printed poster, an audio message, and a brief video presentation (Fig. 1). The intervention materials were developed based on findings from previous research conducted in Ghana by the senior member of the research team [18, 30, 31]. The messages in the materials were based on the Cultural Empowerment domain of the PEN-3 Model [32]. Positive, existential, and negative beliefs about cervical cancer risk factors and cervical cancer prevention were the focal points of the messages. For example, messages addressed cultural beliefs related to the causes of cervical cancer, for example, cervical cancer is caused by a curse.

The poster contained messages focused on aimed at increasing awareness about cervical cancer risk factors and the importance of early detection. The audio message was recorded in Twi and contained messages aimed at promoting the early detection of cervical cancer and debunking myths about cervical cancer risk factors. The video presentation used a photomatic format. The video was narrated in English by a Ghanaian. The messages in the video were focused on increasing awareness about HPV and the risk factors for cervical cancer. Participants were asked to complete a set of questionnaires after reviewing each of the materials.

The participants’ general reaction to the intervention material was assessed using a 5-point Likert scale. The responses ranged from “I like it very much” to “I do not like it at all.” A 3-point Likert scale ranging from “Agree” to “Disagree” was used to assess the participants’ thoughts about the following attributes of the intervention material: interesting, attention-getting, direct/to the point, useful information, and relatable. Close-ended questions that allowed participants to select multiple answers were used to determine whether the materials were able to effectively communicate the main point of the cancer education messages. The ability of the materials to motivate the participants to engage in cervical cancer prevention behaviors or seek additional information about cervical cancer screening was also assessed using a combination of close-ended and open-ended questions.

### Data Analysis

Analysis of the data was done using IBM SPSS software for windows, Version 29.0. Descriptive statistics were computed for the responses to the close-ended questions and the sociodemographic data. The research team used a qualitative approach to assess the responses to the open-ended questions. The qualitative data was coded as favorable or unfavorable. There are no standards or guidelines to determine the appropriateness of health communication messages; therefore, the research team deemed the message to be appropriate or effective if the majority of participants (51% or more) responded favorably.

Ethical approval to conduct the study was received from the Ghana Health Service Ethics Review Committee, and the Institutional Review Board at the research team’s university in the United States. Verbal consent was obtained from each participant before participating in the study. Each participant received 100GHC ($10 USD) as honorarium for participating in study.

## Results

### Demographic Characteristics of the Participants

A total of 60 participants who met the eligibility criteria completed the message testing questionnaires for the intervention materials. The demographic characteristics of the participants are displayed in Table 1. The mean age of the participants was 30.6 years old. The majority of the participants (78.3%) were females. Most of the participants were nurses (42.9%), physicians (17.9%), or physician assistants (10.7%).

**Table 1:** Demographic characteristics of the participants

Demographic characteristic	*n* (%)
Age, mean ± SD	30.58 ± 5.014
Type of healthcare provider	
Nurse	24 (42.9%)
Physician	10 (17.9%)
Physician Assistant	6 (10.7%)
Health assistant	2 (3.3%)
Other	4 (6.7%)
Midwife	5 (8.3%)
Pharmacy assistant	5 (8.3%)
Marital status	
Single	37 (61.7%)
Married	21 (35.0%)
Cohabitation	2 (3.3%)
Ethnic group	
Akan	34 (56.7%)
Ewe	10 (16.7%)
Ga	11(18.3%)
Hausa and other northern tribe	4 (6.7%)
Other southern tribe	1 (1.7%)
Religion	
Christian	59 (98.3%)
Muslim	1 (1.7%)
Gender	
Female	47 (78.3%)
Male	13 (21.7%)

### Poster

Approximately 82% of the participants liked the poster, half of whom said that they liked it very much. Nearly 87% of the participants found the messages on the poster to be motivating. Over 70% of participants were either motivated to get a cervical cancer screening or to learn more about cervical cancer. Four out of the 8 female participants who did not find the messages to be motivating said that the poster had good information, but they did not feel the need to take any action. Most of the female participants (91.5%) said that they were likely or somewhat likely to get cervical cancer screening, or to tell someone to get cervical cancer screening after viewing the poster. Over 78% of the participants agreed that the message on the poster had useful information and was direct/to the point, interesting, and attention-getting. Most of the female participants (86.4%) said that the message on the poster was related to them. Most of the participants were able to identify the main point of the poster. For example, 80% of the participants said that the main point of the poster is that every woman should have a cervical cancer screening, and 50% said that cervical cancer is preventable. Less than 10% of the participants said that the message on the poster was confusing. Some (3.3%) said the poster was not clear, and others (5.0%) were either confused about the images on the poster, or about certain facts such as the age at which a woman should begin getting screened.

### Audio Message

Most of the participants (95%) liked the audio message, and more than half of whom (56.7%) said they liked it very much. All participants said the audio message was motivating. Most of the female participants indicated that the audio message motivated them to get a cervical cancer screening (80%) or motivated them to learn more about cervical cancer (66.7%). Most of the female participants (80.9%) said that they were either very likely, or somewhat likely (17.0%), to get screened or tell someone to get a cervical cancer screening after listening to the message. The majority of the participants (94.8%) agreed that the audio message was interesting, direct/to the point, attention-getting, and had useful information. Over 91% of the female participants agreed that the message was related to them. Over 96% of participants said there was nothing confusing in the audio message. Less than 2% of the participants were confused in general. For example, one participant stated that the message on the mode of getting infected with cervical cancer was not clear.

### Video Lesson

Most of the participants who liked the video lesson (73.3%) said that they liked it very much.

All the participants said that they understood the terms used in the instructions for getting a cervical cancer screening. Almost all the participants (96.6%) agreed that the video presented good medical information. More than half of the participants (65%) said that some of the information in the video was new to them, while 10% of the participants said all the information was new to them. Almost all the participants (96.7%) said the video had very useful information. More than half of the participants (57.1%) said that the pictures in the video reminded them of people they know. Over 85% of the female participants said that they were very likely to get screened or tell someone about cervical cancer screening after viewing the video. While a few participants (about 5%) said that the video had too much information, the majority of participants (96.7%) found nothing confusing about the video.

## Discussion

The cervical cancer education intervention materials that we developed were tailored to be culturally specific to the cervical cancer screening barriers that were identified in previous studies [30, 18, 31]. Our assessment of the participants’ general reactions to the intervention materials indicated that the majority of the participants believed that the messages in the poster, the video, and the audio recording were motivating. Nearly all of the participants were able to accurately identify the main point of the messages that were tested. The majority of the female participants indicated that the messages motivated them to get a cervical cancer screening or tell someone about it. Our findings are aligned with the results of other studies aimed at evaluating the effectiveness of culturally appropriate cancer education interventions. For example, Martei and her colleagues [33] found that culturally appropriate video narratives of Batswana breast cancer survivors had a high degree of acceptability and usability among breast cancer patients.

Based on frameworks for developing culturally sensitive health communication messages, we included value-based messages and images of people of African descent in the intervention poster and video [24, 25]. The central value-based messages in the intervention materials were aimed at debunking myths about cervical cancer and increasing knowledge about the importance of cervical cancer screening. Only a few participants in our study found the messages to be confusing. However, some participants indicated that the images of people did not look like people they know. Further probing of responses to that question suggests that the question was misconstrued by the participants. While we wanted to know if the pictures were representative of Ghanaian people, some participants thought that we were asking “Are these pictures of people you actually know?” Surface structure is a dimension of cultural sensitivity that is centered on increasing the face validity of health communication messages by including observable characteristics of a population [25]. The results of a prior study conducted by Singelis et al. [22] indicate that surface structure has an independent effect on the effectiveness of health communication messages. Therefore, further investigation into the acceptability of the surface structure of our cancer education materials is warranted.

### Implications

Cervical cancer is a leading cause of cancer death among women in Ghana and other LMICs [34]. In 2020, the World Health Organization (WHO) launched an initiative aimed at eliminating cervical cancer globally by 2050 [35]. Experts suggest that increasing adherence to the WHO cervical cancer prevention guidelines for women in LMICs is a cost-effective way to avert DALYs due to cervical cancer [35–38]. Cervical cancer education interventions have shown promising results in some LMICs [20, 28]. Culturally sensitive cervical cancer education interventions should be a component of innovative multi-level strategies in order to have the greatest impact on the increasing adherence to cervical cancer prevention recommendation among women in Ghana and other LMICS [19, 39]. The findings of this study suggest that there is a need for cervical cancer education materials that are targeted towards the multiple cultural deep structure and surface structure characteristics of Ghanaian women.

### Limitations

A strength of this study was that we included a variety of different types of healthcare providers to ensure that our sample was representative of the different types of healthcare providers in Ghana. We also included female and male healthcare providers, since male healthcare providers should be engaging their patients in shared decision making regarding cervical cancer screening. One limitation of the study is the possibility of social desirability bias. Another limitation of our study is that all of participants worked in the Greater Accra Region of Ghana. Therefore, the results of this study may not be transferable to people in other contexts.

## Conclusion

Cervical cancer education interventions are an important component of multi-level strategies for eliminating cervical cancer in LMICs. Our culturally sensitive cervical cancer education intervention materials were found to be acceptable and effective at conveying cervical cancer prevention messages. The value-based messages were effective at motivating female participants to get a cervical cancer screening or tell someone about it. Further investigation into the effectiveness of the surface characteristics of the intervention materials is needed to ensure that the Ghanaians are properly represented in the intervention materials.
